# Stability of Alkyl Chain-Mediated Lipid Anchoring in Liposomal Membranes

**DOI:** 10.3390/cells9102213

**Published:** 2020-09-29

**Authors:** Lukas Gleue, Jonathan Schupp, Niklas Zimmer, Eyleen Becker, Holger Frey, Andrea Tuettenberg, Mark Helm

**Affiliations:** 1 Institute of Pharmaceutical and Biomedical Science, Johannes Gutenberg-University Mainz, 55128 Mainz, Germany; lugleue@uni-mainz.de; 2 Department of Dermatology, University Medical Center, Johannes Gutenberg-University Mainz, 55131 Mainz, Germany; jonschup@uni-mainz.de (J.S.); Niklas.Zimmer@unimedizin-mainz.de (N.Z.); 3 Department of Chemistry, Johannes Gutenberg-University Mainz, 55128 Mainz, Germany; ebecker@uni-mainz.de (E.B.); hfrey@uni-mainz.de (H.F.)

**Keywords:** liposomes, click chemistry, polyglycerol, bioconjugates, drug delivery

## Abstract

Lipid exchange among biological membranes, lipoprotein particles, micelles, and liposomes is an important yet underrated phenomenon with repercussions throughout the life sciences. The premature loss of lipid molecules from liposomal formulations severely impacts therapeutic applications of the latter and thus limits the type of lipids and lipid conjugates available for fine-tuning liposomal properties. While cholesterol derivatives, with their irregular lipophilic surface shape, are known to readily undergo lipid exchange and interconvert, e.g., with serum, the situation is unclear for lipids with regular, linear-shaped alkyl chains. This study compares the propensity of fluorescence-labeled lipid conjugates of systematically varied lengths to migrate from liposomal particles consisting mainly of egg phosphatidyl choline 3 (EPC3) and cholesterol into biomembranes. We show that dialkyl glyceryl lipids with chains of 18–20 methylene units are inherently stable in liposomal membranes. In contrast, C16 lipids show some lipid exchange, albeit significantly less than comparable cholesterol conjugates. Remarkably, the C18 chain length, which confers noticeable anchor stability, corresponds to the typical chain length in biological membranes.

## 1. Introduction

Liposomes are spherical nanovesicles consisting of lipids, which were first produced in the 1960s by Alex Bangham et al. [1]. Due to the amphiphilic properties of phosphatidyl choline lipids in combination with cholesterol-based lipids, liposomes possess an aqueous core and one or more hydrophobic phospholipid bilayers. This enables the incorporation of hydrophobic molecules into the membranes as well as the transport of water-soluble molecules in the core compartment. Because of these properties, liposomes have found application in cancer treatment, gene therapy, cell biology and as a powerful drug delivery system [2,3,4,5,6,7,8,9,10].

Phospholipids, glycerophospholipids and derivatives of cholesterol can be used for the production of liposomes, and this variety of lipids allows the formulation of liposomes with different properties, especially adapted to the respective application.

For example, by adding cholesterol up to 50 mol-% to a liposome formulation, membrane stability and tightness can be increased or particle uptake by macrophages can be reduced by using lipids with linked polyethylene glycol (PEG) chains [11]. These commonly called “PEGylated” liposomes show peculiar behavior, known as the “stealth effect”, i.e., significantly increased blood circulation half-life [12,13]. This effect results from shielding by the long polyethylene glycol chains, which reduces the binding of blood plasma components and interaction with macrophages, leading to increase half-life in blood circulation [14,15]. In addition to the classic example of PEGylation, the conjugation of liposomes to antibodies, folic acid, biotin or peptides can be used for specific tumor targeting [16,17,18,19,20].

We have previously established hyperbranched polyglycerol (*hbPG*)-derived stealth-like polyether lipid structures, possessing either a dialkyl or cholesterol anchor [21]. In human blood serum, liposomes containing hyperbranched polyglycerols showed lower aggregation than comparable PEGylated liposomes by comparable biodistribution [22,23]. Furthermore, hbPG-functionalization leads to increased interaction with macrophages, probably triggered by the lower amount of adsorbed proteins on the surface compared to classical PEGylated particles. This effect might possibly result from the larger number of free hydroxy groups in hbPG-functionalized liposomes, although experimental verification of this hypothesis is not yet available [5].

Cholesterol and many of its metabolic derivatives (such as steroid hormones) are distributed in the bloodstream by lipid–protein particles of variegated density. In addition to receptor-mediated uptake, steroid structures are thought to insert into, or pass through, cell membranes in the frame of a dynamic equilibrium [24,25]. Hence, lipid extraction from liposomes in a similar equilibrium is quite relevant for the sustained properties of liposomal formulations. Previous research of our group established the monitoring of lipid escape from the liposomal membrane and insertion into other membranes. This behavior was particularly evident for cholesterol lipids, which, presumably due to their irregular scaffold structure, are less stably anchored in membranes than typical biomembrane lipids whose two long hydrocarbon chains are known to form a dense network of van-der-Waals interactions in a symmetric lipophilic environment such as a cell membrane [26]. In order to demonstrate this effect, we prepared liposomes based on functionalizable cholesterol and dialkyl lipid derivatives, to which fluorescent dyes were covalently attached by click chemistry. After 4 h incubation of cells with the fluorescently labelled liposomes, confocal laser scanning microscopy images showed a significant fluorescence of labeled cholesterol derivatives in the cell membrane, in contrast to labeled dialkyl lipids which rarely inserted into the cell membrane [25].

The choice of appropriate lipid anchors can therefore influence a number of important parameters of liposomal formulations, e.g., the sustained transport (or release) of a model cargo during extended circulation, or the durability of molecular structures conjugated to the liposomal surface, which are important for stealth shielding or for targeting to specific receptors involving molecular recognition of bioconjugates [27,28].

In the present study, we identify a lower limit for dialkyl chain length in lipids, which leads to the exchange between the liposomal membrane and the cell membrane.

## 2. Materials and Methods

### 2.1. Synthesis of Dialkyl-Based Anchors

Three different hydrophobic anchor structures were synthesized for use as initiators for the anionic ring-opening polymerization (AROP) of epoxides. The initiators were prepared in a straightforward two-step procedure based on a procedure of Stauch et al., performing a Williamson etherification and using 1-*O*-benzyl glycerol and hexadecyl bromide, octadecyl bromide as well as icosane bromide, followed by hydrogenation to remove the benzyl protecting group [29]. In order to investigate the influence of the chain lengths, the anchors 1,2-bis-*n*-icosanyl glyceryl ether (BisID), 1,2-bis-*n*-hexadecyl glyceryl ether (BisHD) and 1,2-bis-*n*-octadecyl glyceryl ether (BisOD) were synthesized. The alkyl chain lengths of the anchor structures were 20 (BisID), 18 (BisHD) and 16 (BisOD) carbon atoms.

### 2.2. Synthesis of Amphiphilic Polyethers

The anionic ring-opening polymerization (AROP) of ethylene oxide was applied to synthesize well-defined PEG chains with tailored molecular weights [30]. The 1,2 bis-*n*-alkyl glyceryl ethers were used as initiators for the polymerization to obtain amphiphilic polyether-based lipids (see supplement scheme S1). The combination of a hydrophobic initiator and a hydrophilic polyether results in polymers with amphiphilic behavior, which are suitable for the integration into liposomes to obtain sterically stabilized liposomal nanocarriers.

The AROP for the polymerization of ethylene oxide (EO) was carried out in dry tetrahydrofuran (THF), and potassium naphthalenide was used as a base to deprotonate the initiator. The deprotonation process was followed by color change of the initiator solution. The polymerization was carried out at 60 °C for 24 h to obtain full conversion. Molecular weights are controlled via the ratio of employed initiator and epoxide monomer.

### 2.3. Functionalization of Amphiphilic Polyethers with Propargyl Bromide

**Figure 1:**
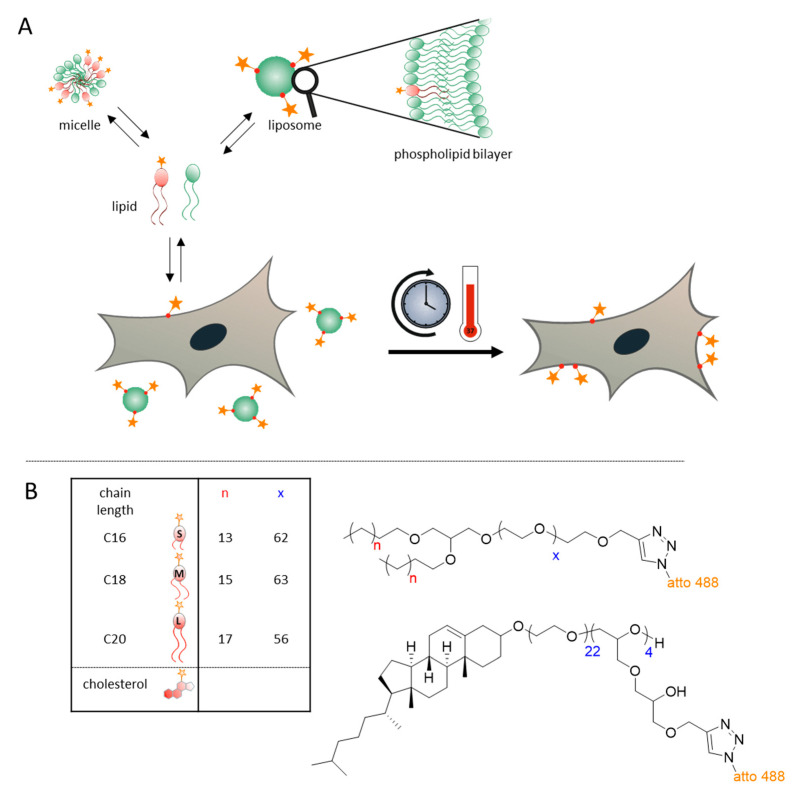
(**A**) Conceptual sketch of the approach. Lipid compounds are distributed among a variety of lipid-containing bodies in a complex equilibrium with ill-defined dynamics. The star symbolizes the attached fluorescence dye atto 488. (**B**) Synthetic experimental polyether lipids employed in the investigations: n describes the number of carbon atoms in alkyl chains, x the number of polyethylene glycol (PEG)-units.

To attach alkyne-moieties, the polyether-based lipids were functionalized with propargyl bromide at the polyether end group. An alkyne-group enables the copper(I)-catalyzed azide-alkyne cycloaddition (CuAAC) with functional groups, e.g., dyes like atto 488 azide or other azide-bearing molecules [25]. This reaction is known from literature and was adapted for the synthesized amphiphilic polyethers [31,32]. For this purpose, the terminal hydroxyl group of PEG was deprotonated using sodium hydride (NaH) (see supplement scheme S1). The terminal propargyl group was then introduced by a substitution reaction with propargyl bromide (structures: Figure 1B).

### 2.4. Synthesis of Cholesterol-Based Linear-Hyperbranched Polyethers

As a drawback, despite its combination of favorable properties, such as excellent aqueous solubility, low toxicity and biocompatibility, PEG exhibits the disadvantage of low functionality, i.e., only the terminal hydroxyl groups [30,33]. Hyper-branched structures based on polyglycerol (*hb*PG) are therefore a promising alternative, due to the multitude of hydroxyl groups available for post-polymerization modification. Furthermore, *hb*PG is highly water-soluble and also exhibits excellent biocompatibility [34]. Hofmann et al. introduced amphiphilic *hb*PGs using different hydrophobic initiators (cholesterol, 1,2-bis-*n*-tetradecyl glyceryl ether (BisTD), 1,2-bis-*n*-hexadecyl glyceryl ether (BisHD) and 1,2-bis-*n*-octadecyl glyceryl ether (BisOD)) [21,35].

In the first reaction step a cholesterol-PEG-PEEGE precursor polymer was synthesized via AROP with EO and ethoxyethyl glycidyl ether (EEGE) (see supplement scheme S2). The polymerization of EEGE leads to linear structures (PEEGE) with acetal-protected hydroxyl groups, which can be released upon acidic treatment. The resulting linear polyglycerol (*lin*PG) structure exhibits numerous hydroxyl groups and can therefore be used as a macroinitiator to prepare a hyperbranched polyglycerol (*hb*PG) block. Consequently, after the deprotection under acidic conditions, the cholesterol-PEG-*lin*PG macroinitiator was used for “hypergrafting” of glycidol via the slow monomer addition (SMA) technique to obtain the cholesterol-PEG-*hb*PG polyether lipids (Figure 1B).

### 2.5. Functionalization of Cholesterol-PEG-hbPG with Propargyl Bromide

Polymer derivatization for subsequent functionalization by click chemistry was carried out using propargyl bromide. The hydroxyl groups of *hb*PG were deprotonated using sodium hydride (NaH). In the case of the functionalization of *hb*PG, the degree of functionalization was controlled via the amount of propargyl bromide employed. The average degree of functionalization was determined via ^1^H NMR spectroscopy [29].

#### 2.5.1. Liposome Preparation

In the first step, for the preparation of 5 mM liposomes, lipids dissolved in chloroform (see Table 1) were added to a PCR tube (#G001 F, Kisker Biotech, Steinfurt, Germany) depending on their percentage of total lipid amount and the solvent was removed by vacuum centrifugation (#5305, Eppendorf, Hamburg, Germany).

For complete removal of solvents, lipids were then freeze-dried at −80 °C (Alpha 2–4 LD, Martin Christ Gefriertrocknungsanlagen, Osterode/Harz, Germany) overnight and stored in a freezer until use at −20 °C.

To prepare liposomes, dried lipids were incubated together with 325 mg SiLibeads (#96035, Typ ZY-S 0.3–0.4 mm, Sigmund Lindner, Warmensteinach, Germany) and 9.3 µL DPBS (Dulbecco’s phosphate-buffered saline (#14190-094, Thermo Fisher Scientific, Waltham, MA, USA)) for 10 min at room temperature and then mixed for 20 min at 4 °C by dual centrifugation (#3200 + #3205, Andreas Hettich, Tüttlingen, Germany) to form a phospholipid gel. To finally form liposomes, the gel was homogenized again together with 77.2 µL DPBS twice for 2 min at 4 °C.

#### 2.5.2. Liposome Modification and Purification

To label alkyne liposomes with fluorescent dye atto 488-azide, 30 µL liposome stock was dissolved in 100 mM phosphate buffer pH 8 (PB) (94.7 mM disodium hydrogen phosphate (#P030.1, Carl Roth, Karlsruhe, Germany), 5.3 mM sodium dihydrogen phosphate dihydrate (#T879.1, Carl Roth)), 0.5 mM Tris (hydroxypropyltriazolylmethyl) amine (THPTA, Helm Group, Johannes Gutenberg University Mainz, Germany), 0.1 mM copper sulfate pentahydrate (#8175.1, Carl Roth,), 2.5 mM soduim ascorbate (#3149.1, Carl Roth), 0.1 mM atto 488-azide (#AD 488-101, Atto-Tec, Siegen, Germany) in a PCR tube (#G001 F, Kisker Biotech) and filled up with MiliQ-Water (#ZRQSVR5WW, Merk Millipore, Darmstadt, Germany) to a finale volume of 120 µL.

The reaction mixture was agitated at room temperature for two hours and after completion of the transformation, 20 mM EDTA (ethylenediaminetetraacetic acid (#8040.1, Carl Roth, solved in water)) was added to stop the reaction.

#### 2.5.3. Cell Culture and Cell Lines Employed

The human melanoma cell line UKRV-Mel-15a was cultured in RPMI 1640 medium (#31870, Thermo Fisher Scientific, Waltham, MA, USA) supplemented with 10% FBS (fetal bovine serum (#10500064, Thermo Fisher Scientific)), 1% GlutaMAX^TM^ (#35050038, Thermo Fisher Scientific) and 0.1% primocin (#ant-pm-2, InvivoGen, San Diego, CA, USA). Cells were detached via incubation Trypsin-EDTA (#T3924, Merck, Darmstadt, Germany) for 5 min at 37 °C every 3 to 4 days.

#### 2.5.4. Flow Cytometry

For analysis of lipid exchange via flow cytometry UKRV-Mel-15a cells were seeded in 24 well tissue culture plates (#3524, Corning, Corning, NY, USA) at a density of 100,000 in 1 mL medium and incubated for 4 or 24 h with liposomes at a concentration of 1% or 5% (*v*/*v*). For Section 3.5, adherent cells were treated additionally with 10 µM cytochalasin D (#C2618, Merck) 30 min prior to liposome addition [36,37,38]. Cells were detached via incubation with Trypsin-EDTA (#T3924, Merck)) for 5 min at 37 °C. Singe cell suspension was stained with 200fold diluted Fixable Viability Dye eFluor™ 780 (#65-0865-18, Thermo Fisher Scientific) in DPBS (#14190-094, Thermo Fisher Scientific) for 20 min at 4 °C for dead cell exclusion, fixed with 4% PFA (paraformaldehyde (#0335.1, Carl Roth)) in DPBS for 20 min at 4 °C and measured in FACS buffer containing 0.5% HSA (#10530a/96, CSL Behring, Marburg, Germany), 1 mM EDTA (#A3553, AppliChem, Darmstadt, Germany), 10 μg/mL human IgG (#EU/1/08/446/001, CSL Behring GmbH, Marburg, Germany) in DPBS.

Flow cytometry was performed on an LSRII flow cytometer (BD Biosciences, Franklin Lakes, NJ, USA) and samples were measured by 488 nm (FITC-channel 505LP, 530/30) and by 633 nm (APC-channel 660/20; FVD780 735LP, 780/60). Data were analyzed by using Cytobank [39].

#### 2.5.5. Microscopy

For the confocal imaging, the Leica SP8 with HyD Detector (Wetzlar, Germany) was used with lasers for 405 nm, 488 nm and 638 nm. Melanoma cell line UKRV-Mel-15awas cultured for 24 h in ibidi µ-slides 8 well (#80826, Ibidi, Gräfelfing, Germany), 30,000 cells/well each, in supplemented RPMI 1640 medium (see cell culture cell line). After treatment for 4 and 24 h with the same endpoint, cells were checked for adherence and then fixed with 4% PFA (#0335.1, Carl Roth, Karlsruhe, Germany) in DPBS for 20 min at 4 °C. Additionally DNA was stained by Hoechst 33342 (#PK-CA707-40046, PromoCell, Heidelberg, Germany)) and the membrane by DiD (1,1-Dioctadecyl-3,3,3,3-tetramethylindodicarbocyanine (#PK-CA707-30021, PromoCell, Heidelberg, Germany)) for 30 min at RT each. For image analysis, Fiji was used [40].

#### 2.5.6. Dynamic Light Scattering

For liposome characterization, 20 µL purified liposome suspension was diluted in 1 mL MiliQ-water and polydispersity (PDI), size and zeta-potential (ζ-potential) were measured in a disposable folded capillary cell (#dts1070, Malvern, Worcestershire, United Kingdom) at 25 °C by using a Malvern Zetasizer Nano series (Malvern). The refractive index was set to 1.33 (1.59 for liposomes) with a water viscosity of 0.8872 and the scattering angle was configured to 173.

## 3. Results

### 3.1. Concept and Approach

The strategy for monitoring of the fate of different dye-conjugated lipids is shown in Figure 1A. The upper part depicts in a general manner the behavior of single lipid molecules in equilibrium between micelles, free lipids and liposomal structures. In in vitro cell culture, the latter also includes the cell membranes, which participate in an exchange equilibrium with liposomes.

To monitor the exchange equilibrium, we labelled the polyether model lipids with a fluorescent dye and traced their location both by microscopy and by flow cytometry. Suspension of liposomes containing fluorescence-labelled lipids were added to cell cultures, and after a certain incubation period, the localization and intensity of fluorescence in the cell membranes were analyzed. Of note, we are aware that we cannot directly distinguish between lipid exchange taking place at the cell membrane with liposomes in suspension or phagocytosis of liposomes and their subsequent degradation and incorporation into the cell membrane system. However, time-lapse imaging with this approach has previously enabled us to efficiently monitor the fast exchange of cholesterol derivatives between liposomes and cell membranes [26]. We observed a rapid exchange rate in the single-minutes digit for cholesterol derivates, with fluorescent lipids first appearing in the cell membrane and only later being incorporated into intracellular vesicles [26]. In this work, we observed a significantly slower exchange for alkyl chains up to a time of 4 h, leading us to conduct the current observation at 4 h and 24 h. The latter time point was chosen to obtain a measure of the duration of the lipid exchange. To guard against the potential adverse effects of endocytosis, we also performed experiments in the presence of cytochalasin D, an endocytosis inhibitor.

### 3.2. Experimental Lipids and Liposome Preparation

Figure 1B depicts the different compounds employed in this setting, which were synthesized based on our prior discovery that revealed high dynamics of a cholesterol derivative in transitions from liposomal preparations to cell membranes [26]. The model compounds employed are conceptually similar to biological membrane lipids containing two long alkyl chains as well as a hydrophilic headgroup, which in the current work is a polyethylene glycol chain (or alternatively a *hb*PG structure) that additionally equips the liposomes with a stealth effect. The synthesis of these compounds was conducted along the lines described previously by Frey et al. [21,23,35]. Briefly, the synthesis of PEGylated lipids was performed by anionic ring-opening polymerization (ROP) starting from dialkyl-substituted glycerol. Subsequent to the polymerization of EO, the resulting PEGylated ether lipids were reacted with propargyl bromides to functionalize the chain ends with a propargyl-ether.

The terminal alkyne moieties allowed for subsequent derivatization by click chemistry either before (pre) or after (post) liposomal formulation. Our current investigation relies on post-formulation CuAAc of liposomes with fluorescent dyes, which were then purified by gel filtration chromatography. The advantages of this formulation–derivatization sequence include mild reaction conditions and fast product isolation by routine semi-automated methods [41].

The production of liposomes was conducted by dual centrifugation, a technique based on the simultaneous rotation of two sample plates around two axes. This results in the mixing of the samples instead of sedimentation as in classical centrifugation. The advantages of using dual centrifugation to produce liposomal formulations are the choice of flexible preparation sizes with simultaneous sterile and endotoxin-free conditions. In addition, this cost-effective method allows for the simultaneous preparation of several samples [26,42].

The liposome suspensions prepared by dual centrifugation were then fully automatically purified by using semi-automated gel filtration on an HPLC (high-performance liquid chromatography) setup to remove free lipids. Routine characterization included size determination by dual light scattering and measurement of surface potential. For this work, we used liposomes with 162.60 ± 29.51 nm diameter, 0.26 ± 0.07 a.u. polydispersity and −13.87 ± 0.07 mV zeta-potential.

### 3.3. Analysis of Lipid Exchange Via Flow Cytometry

To compare the transfer of fluorescently labelled experimental lipids from liposomes to cells in tissue culture, cells were cultivated for 4 h and 24 h with liposomal formulations containing the fluorescence-labeled lipids in two different concentrations. In comparison to previously investigated RBE4 cells [26], we found the human melanoma cell line UKRV-Mel-15a more amenable to rapid and reproducible analyses, presumably because of their relatively large size. After incubation with liposomal preparations, the medium was removed, cells were fixed and then analyzed by flow cytometry.

**Figure 2:**
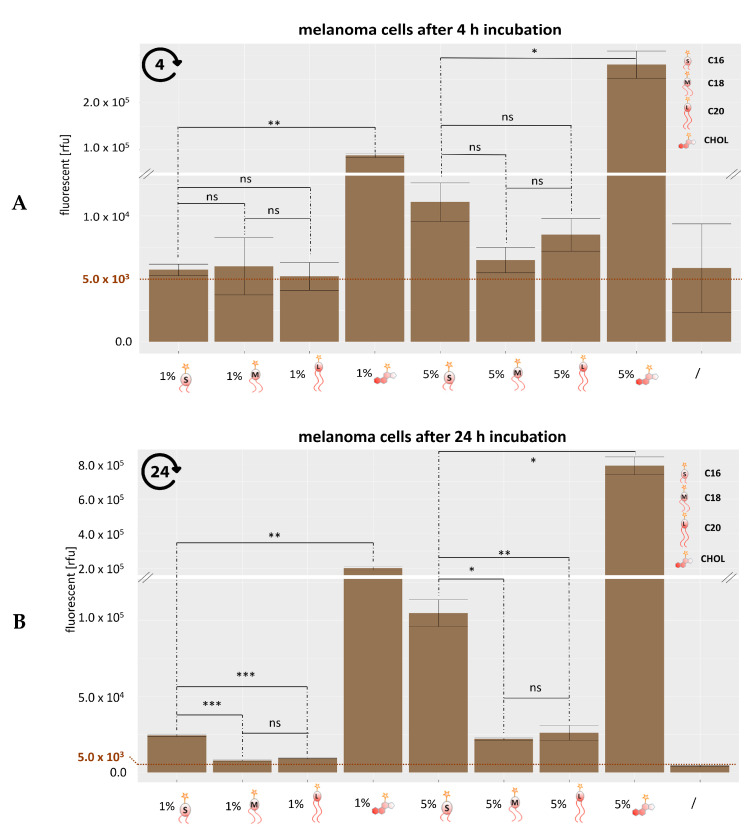
Quantification of lipid exchange after 4 h (**A**) and 24 h (**B**) by flow cytometry. 1.0 × 10^5^ UKRV- Mel-15a cells were incubated with 1 or 5 vol-% atto 488 linked liposomes at 37 °C and fixed with a 4% formaldehyde solution before flow cytometric analysis. The significance between two values is illustrated by * *p* = 0.05–0.01, ** *p* < 0.001, *** *p* < 0.0001 and ns = no significance. The statistically significant was calculated using the Welch and Brown–Forsythe version of the one-way ANOVA test.

The results after gating signals for the atto 488 are presented in Figure 2A,B. Figure 2A, reflecting the fluorescence intensity obtained after an incubation of 4h, clearly reproduces the behavior of cholesterol-anchor derived lipids as previously published, showing strong signals corresponding to a transfer of cholesterol derived lipids to the cell membranes [26]. For the dialkyl lipids, most signals remained near a baseline of 5.0 × 10^3^ with the visually apparent, but not significant, exception of the liposomal preparation containing 5 mol-% C16 compounds, i.e., the shortest alkyl chain used in this study. Additionally, cells incubated with C18 and C20 liposomes for 4 h showed moderately, but not significantly, increased fluorescence.

Data obtained after 24 h (Figure 2B) demonstrate the progression of lipid exchange for all preparations containing 5 mol-% polyether lipids, albeit only to a very limited extent for the C18 and C20 anchor chains. Meanwhile, in further confirmation of our previous observations, the signal resulting from the cholesterol derivative outdistanced all others by far, more subtle differences were observable among the polyether lipids.

Thus, the C16 preparations both at 1 mol-% and 5 mol-% now gave rise to significantly higher signals than those of the corresponding longer C18 and C20 alkyl chains. These observations suggest that for the anchoring of stealth structures with alkyl chains, a minimum alkyl chain length of C18 is very efficient, whereas a length of 16 carbon atoms delineates a border, below which anchoring might be insufficient. Future studies including, e.g., C14 and C12 polyether lipids, must be undertaken to understand if chain length is the only determinant of membrane anchorage.

### 3.4. Tracking Labeled Lipids by Fluorescence Microscopy

**Figure 3:**
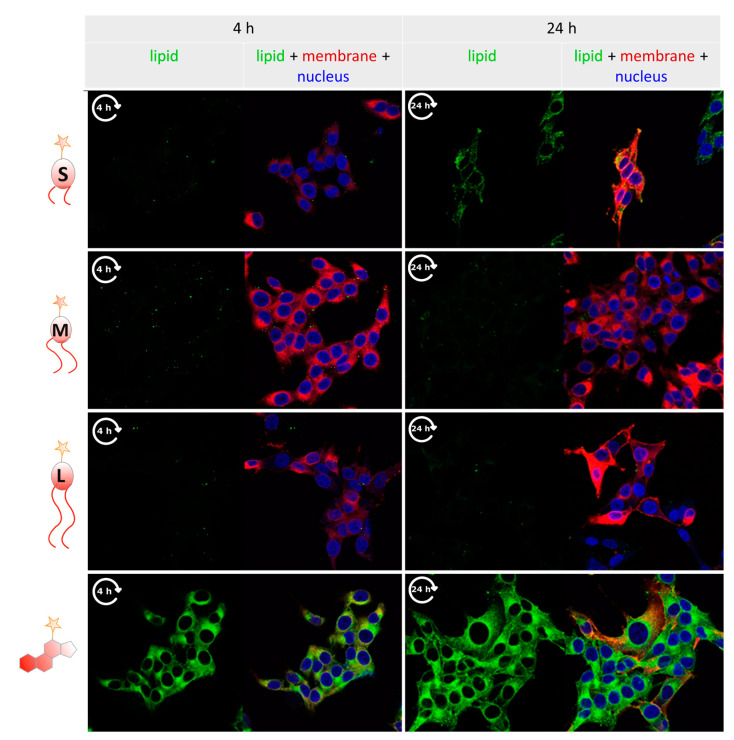
Confocal fluorescence imaging of UKRV-Mel-15a cells, observed at 4 h and 24 h after incubation with liposomes containing membrane label (DiD, red) and click-labeled experimental lipids with varied chain length (atto 488, green). Cell nuclei were stained with Hoechst (blue). Together with the cell membrane shown in red, the insertion of lipids becomes visible as an orange signal in the merge view.

For a more spatially resolved, detailed investigation, cells exposed to liposomes as described above for the flow cytometry analyses were imaged by confocal fluorescence microscopy. The results for 4 h and 24 h incubation at 37 °C are shown side-by-side in Figure 3.

As a positive control for lipid exchange, the liposomes contained a DiD label, which is observed to stain the cells’ biomembranes in the red fluorescence channel clearly. In comparison to this efficient exchange from liposomes to cell membranes, C18 and C20 lipids hardly show signals of relocation, which is coherent with the observations in flow cytometry stated above. In contrast, relocation was easily visible for cholesterol-conjugated dye, which rapidly stained cell membranes, indicated by green fluorescence from the atto 488 dye, or by an overlay of red and green fluorescence visualized in orange in Figure 3, this observation, too, is well in line with the flow cytometry data. To a lesser extent, but clearly visible, the relocalization of green fluorescence was observed for the labeled C16 lipid after 24 h (upper right panel in Figure 3), signaling a certain extent of lipid exchange from liposomes to the cell membrane. In summary, these images thus confirm the tendency of C16 alkyl lipid to enter into a dynamic equilibrium as depicted in Figure 1A in a time frame comprising a few hours to one day.

### 3.5. Analysis of Lipid Exchange Depending on Liposome Composition

To further characterize the differential behavior of C1b versus longer alkyl lipids in some more depth, we investigated the influence of liposome composition on the lipid exchange. Liposomes containing 5 mol-% C16 versus C18 alkyne lipids were prepared with different EPC3: Chol ratios, namely 50/45, 60/35 and 70/25. Lipid exchange after 24 h incubation was analyzed by flow cytometry as described above. To suppress the potential influence of endocytotic uptake, cell culture experiments were carried out in the presence of 10 µM endocytosis inhibitor cytochalasin D.

**Figure 4:**
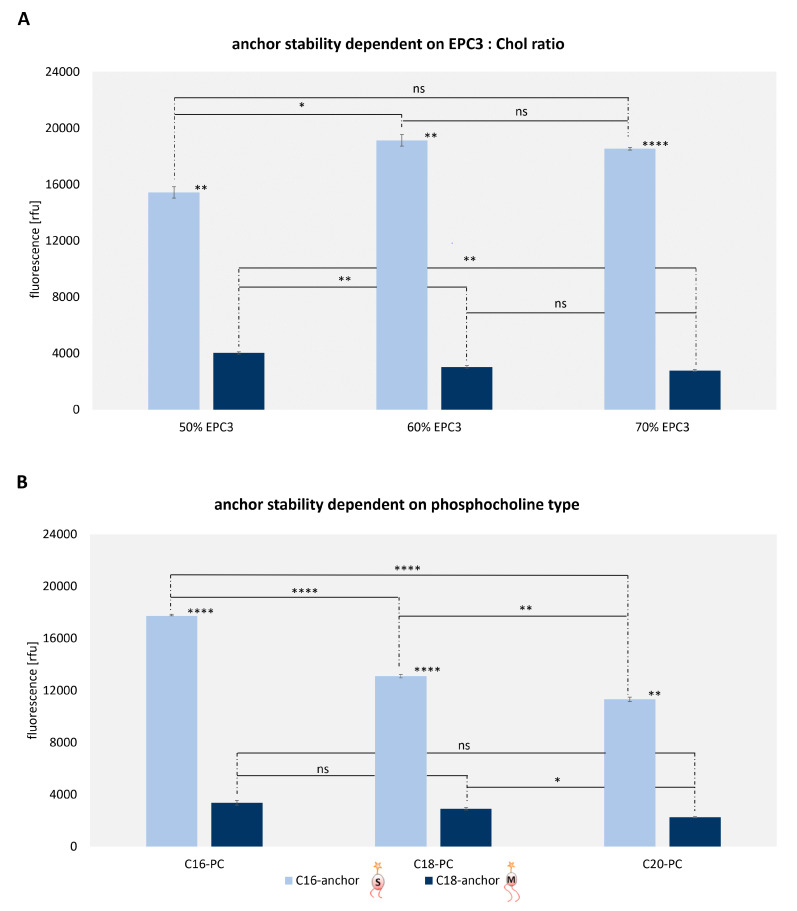
Quantification of lipid exchange depending on (**A**) the EPC3: Chol (cholesterol) ratio (**B**) and on the carbon atom number of phosphocholines used for liposome preparation. 1.0 × 10^5^ UKRV Mel-15a cells were incubated with 5 vol-% atto 488 linked liposomes and cytochalasin D at 37 °C and fixed with a 4% formaldehyde solution before flow cytometric analysis. The significance between two values is illustrated by * *p* = 0.05–0.01, ** *p* < 0.001, **** *p* > 0.0001 and ns = no significance. The statistically significant was calculated using the Welch and Brown–Forsythe version of the one-way ANOVA test.

The results shown in Figure 4A show slightly decreased exchange and thus more stable anchoring for C18 lipids at higher EPC3 content, while the exchange of the C16 lipid peaks at medium EPC3 content. The principle insight obtained here, though, is that the difference between C16 and C18 is highly significant at all ratios, and that C16 maintains similarly high lipid exchange rates even at increased EPC3 content.

In a similar experiment, we investigated the effect of phosphocholines with different dialkyl chain lengths. Correspondingly liposomes were prepared, which contained phosphocholine with chain lengths of C16, C18, or C20 instead of EPC3. Each formulation contained 5 mol-% C16 or C18 alkyne lipid. The results after 24 h incubation shown in Figure 4B suggest a slightly increased anchoring of our C18-hbPG-lipid in membranes containing long-chain phosphocholine, but this visual impression is not statistically significant. Similarly, our C16-hbPG-lipid displays reduced lipid exchange in longer EPC chains, and this effect is highly significant. Throughout, the mobility of the C16 compound is clearly higher than that of the C18 compound.

## 4. Discussion

In the present study, we showed results on the mobility of amphiphilic polyether lipids in liposomal membranes with respect to their propensity to insert into cell membranes in a time frame of up to one day, which is a relevant time span for testing liposomal formulations in either in vitro or in vivo.

We used fast exchanging cholesterol derivatives as a positive control in the comparative analysis of a mini-series of homologous lipids containing dialkyl chains of increasing length [26]. The data firmly established that dialkyl anchors of any tested length are much more stably anchored in the liposomal membrane than cholesterol derivatives, extending previous observations to lipids as short as C16 [26]. This is plausible, given the irregular shape of cholesterol, yet interesting with respect to the behavior of biological lipids with chain lengths shorter than the classical C18. Of interest, the lipids forming the liposomal double-membrane consisted of nearly equal parts cholesterol, and phosphatidylcholine, the latter being structurally more similar to the dialkyl lipids. Although the lipid-dye conjugate used here to trace the lipid exchange contained a rather hydrophilic polyglycerol chain, which is likely to enhance its extraction from a membrane, our results support a view of “aging” liposomes, which may dramatically change their composition when exposed to a “lipid extraction equilibrium” as depicted in Figure 1B. This may give rise to disadvantages on multiple levels [43,44]. One such level is the structural integrity of the liposome, required to retain or release its therapeutic cargo in a reproducible fashion. Clearly, this important property may be subject to dramatic alterations, depending on the lipid extraction conditions in vivo, but also depending on the propensity of a given liposomal constituent compound for lipid exchange. While previous work identified cholesterol conjugates as problematic, our current data offer an approximation for the choice of non-steroid lipids capable of reducing the risk of undesired lipid exchange [26].

On another level, the molecular surface of liposomes, especially when decorated with targeting or stealth structures, is likely to suffer from lipid exchange. Here, too, our studies provide important results. In particular, we show that dialkyl lipids with 16C chains are more likely to escape from liposomal membranes than longer-chain lipids. This behavior may be important for the choice of dialkyl lipids as anchors for modifications, such as PEGylation or antibody conjugation, because the number of carbon atoms in dialkyl chains may have an influence on stability and half-life time of a modification in the application of liposomes as drug delivery systems. Particularly after intravenous application, liposomes encounter various moieties capable of lipid exchange, including cellular components of blood such as erythrocytes and thrombocytes, but also various protein–lipid particles involved in lipid transport such as HDLP, LDLP, and the like.

As a final comment, we would like to point out the striking fact that we detect increasing propensity for lipid exchange for lipids with alkyl chains just below the typical length encountered in many biomembranes, namely C18 (e.g., oleic acid, vaccenic acid or stearic acid) [45,46]. While ours is but a single data point of one lipid with C16 chains, it might be worth looking into this border in a more systematic fashion.

## Figures and Tables

**Table 1:** Lipid compositions of liposomal formulations in mol-%.

Lipid 1	Percentage	Lipid 2	Percentage	Lipid 3	Percentage
EPC3	50 mol-%	Cholesterol	45 mol-%	C16 or C18 or C20 or Cholesterol alkyne lipid	5 mol-%
EPC3	60 mol-%	Cholesterol	35 mol-%		
EPC3	70 mol-%	Cholesterol	35 mol-%	C16 or C18 alkyne lipid	5 mol-%
DPPC	50 mol-%	Cholesterol	45 mol-%		
DSPC	50 mol-%	Cholesterol	45 mol-%		
20:0 PC	50 mol-%	Cholesterol	45 mol-%		

EPC3: egg phosphatidyl choline 3 (#527600, lipoid, Ludwigsharfen, Germany); DPPC: 1,2-dipalmitoyl-sn-glycero-3-phosphocholine (#850355C-200MG, Sigma-Aldrich, St. Louis, MO, USA); DSPC: 1,2-distearoyl-sn-glycero-3-phosphocholine (#850365C-200MG, Sigma-Aldrich); 20:0 PC: 1,2-diarachidoyl-sn-glycero-3-phosphocholine (#850368C-25MG, Sigma-Aldrich).

## Supplementary Materials

The following are available online at https://www.mdpi.com/2073-4409/9/10/2213/s1, Scheme S1: Synthesis Route of the Dialkyl PEG-Lipids and Functionalization with propargyl bromide. Scheme S2: Synthesis Route of the Cholesterol-PEG-hbPEG Lipids and Functionalization with propargyl bromide.
