# Supplementary material for: Stability of Alkyl Chain-Mediated Lipid Anchoring in Liposomal Membranes
Source: Cells. 2020 Sep 29;9(10):2213. doi: 10.3390/cells9102213 (PMC7599733; doi:10.3390/cells9102213)
Supplement: Supplementary file 1 [file cells-09-02213-s001.pdf]

cells MDPI

Supplementary Material

S1. Materials and Methods

S1.1. Reagents

Unless mentioned otherwise, all chemicals were obtained from *Sigma Aldrich*, *Acros Organics*, *Fisher Scientific* or *TCI Europe*. Deuterated solvents (pyridine-*d5*, benzene-*d6* or chloroform-*d*) were purchased from *Deutero GmbH*. Ethylene oxide (EO) was received from *Sigma Aldrich* and must be handled with high precaution. Ethoxyethyl glycidyl ether (EEGE) was synthesized according to literature[1] and was dried over CaH_2 for at least 30 min. Subsequently it was freshly cryo-transferred prior to use. For the anionic ring-opening polymerization (AROP) dry THF was stored over benzophenone/sodium.

S1.2. Instrumentation

^{1}H, ^{13}C NMR and 2D spectra were measured on a Bruker Avance III HD 300 (300 MHz, 5 mm, BBFO probe, and B-ACS 60 auto sampler) or rather a Bruker Avance II spectrometer operated at 400 MHz (5 mm BBFO smart probe and SampleXPress 60 auto sampler) at 296 K. Pyridine-*d5*, benzene-*d6* , chloroform-*d* or DMSO-*d6* was used as a solvent. The NMR spectra were referenced internally to the respective signals of the deuterated solvent. Analysis of all spectra was carried out using the software MestReNova version 9.0.

S1.3. Handling of Ethylene Oxide (EO)

The gaseous, flammable and highly toxic ethylene oxide (EO) must be handled carefully and has to be stored in pressure-proof gas bottles. It must be used only in an adequate fume hood under appropriate safety precautions. Polymerizations in which EO is involved are performed in flame-dried glassware to enable the conversion of EO inside the sealed and evacuated glass apparatus and to guarantee secure handling via cryo-transfer techniques. To avoid abrupt detachment of the septum and hence release of EO the maximum batch-sizes of 8 g EO in a 500 mL flask must not be exceeded.

S2. Synthesis

S2.1. Synthesis of Ethoxyethyl Glycidyl Ether (EEGE)

EEGE was synthesized as described in literature.[1]

^{1}H NMR, COSY (300 MHz, chloroform-*d*, δ): 4.75–4.68 (qd, *J* = 5.3 Hz; 3.4 Hz, 1H, acetal H), 3.86–3.29 (m, 4H, -CHC*H*2OCOCH2-, -CHCH2OCOC*H*2-) 3.13–3.07 (m, 1H, CH2OC*H*CH2-), 2.77–2.74 (ddd, *J* = 5.1 Hz; 4.1 Hz; 1.0 Hz, 1H, C*H*2OCHCH2-), 2.61–2.55 (ddd, *J* = 10.7 Hz; 5.1 Hz; 2.7 Hz, 1H, C*H*2OCHCH2-), 1.29–1.26 (dd, *J* = 5.4 Hz; 4.7 Hz, 3H, -OCHC*H*3), 1.18–1.13 (td, *J* = 7.1 Hz; 0.9 Hz, 3H, -OCH2C*H*3).

S2.2. Synthesis of 1,2-Bis-N-Octadecyl Glyceryl Ether (BisOD)

The synthesis was carried out according to literature.[2]

^{1}H NMR, COSY (400 MHz, chloroform-*d*, δ): 3.74–3.41 (m, 9H, glycerol H), 1.59–1.53 (m, 4H, -OCH2C*H*2-), 1.31–1.25 (m, 60H, -OCH2CH2(C*H*2)15CH3), 0.88 (t, *J* = 13.1 Hz; 7.2 Hz, 6H; -O(CH2)17C*H*3).

^{13}C NMR, HSQC, HMBC (101 MHz, chloroform-*d*, δ): 78.4 (glycerol C), 72.0 (-O*C*H2CH2-), 71.1 (glycerol C), 70.6 (-OCH2CH2-), 63.3 (glycerol C), 32.1 (-OCH2CH2(CH2)17CH3), 30.3 (-OCH2*C*H2-), 29.9–29.5 (-OCH2CH2(*C*H2)17CH3), 26.3 (-OCH2CH2(*C*H2)17CH3), 22.9

(-$OCH_2CH_2(CH_2)_{17}CH_3$), 14.3 (-$O(CH_2)_{19}CH_3$).

S2.3. Polymer Synthesis of BisOD-PEG

The synthesis is described for BisOD-PEG_{81} as a representative example.

1,2-Bis-*n*-octadecyl glyceryl ether (BisOD) (0.2 g, 0.36 mmol, 1 eq.) was placed in a dry Schlenk flask and dissolved in benzene (10 mL). The solution was stirred at 60 °C for 30 min and dried in vacuo for 16 h to remove moisture. Dry tetrahydrofuran (approx. 10 mL) was cryo-transferred to the Schlenk flask to dissolve the initiator. Afterwards, the initiator was deprotonated with a 0.5 M solution of potassium naphthalenide in THF (0.36 mL, 0.18 mmol, 0.5 eq.) while stirring. The solution was cooled down to –80 °C and ethylene oxide (EO) (1.70 mL, 37.57 mmol, 105 eq.) was cryo-transferred using a graduated ampule. The polymerization was carried out at 60 °C for 24 h. In order to quench the polymerization, an excess of ethanol was added. The solvent was removed under reduced pressure, the crude product was dissolved in methanol and precipitated twice in cold diethyl ether to obtain the pure product. Yield: 99%.

^{1}H NMR, COSY (400 MHz, benzene-*d6*, δ): 3.67–3.30 (m, 359H, polyether backbone and glycerol H), 1.66–1.56 (m, 4H, -OCH_2CH_2-), 1.44–1.28 (m, 58H, -$OCH_2CH_2(CH_2)_{15}CH_3$), 0.92–0.89 (m, 6H, -$O(CH_2)_{17}CH_3$).

^{13}C NMR, HSQC, HMBC (101 MHz, benzene-*d6*, δ): 79.0 (glycerol C), 73.4 (polyether backbone and glycerol C), 72.4-70.9 (polyether backbone and glycerol C), 62.2 (polyether backbone and glycerol C), 32.6 (-$OCH_2CH_2(CH_2)_{14}CH_2CH_3$), 31.1–30.1 (-$OCH_2CH_2$- and -$OCH_2CH_2$ $(CH_2)_{14}CH_2CH_3$), 27.0 (-$OCH_2CH_2(CH_2)_{14}CH_2CH_3$), 23.4 (-$OCH_2CH_2(CH_2)_{14}CH_2CH_3$), 14.64 (-$O(CH_2)_{17}CH_3$).

S2.4. Functionalization of BisOD-PEG with Propargyl Bromide

The functionalization is described for BisOD-PEG_{81}-alkyne as a representative example.

BisOD-PEG_{81} (0.3 g, 0.074 mmol, 1 eq.) was placed in a dry Schlenk flask and dissolved in dry THF. The solution was cooled to 0 °C and sodium hydride (0.005 g, 0.223 mmol, 3 eq.) was added while stirring. Subsequently, propargyl bromide (0.020 mL, 0.223 mmol, 3 eq.) was added and the solution was stirred for 24 h at room temperature. The reaction mixture was filtered and the solvent was slightly reduced under reduced pressure. The remaining solution was precipitated twice in cold diethyl ether and the pure product was dried in vacuo. Yield: 66%.

^{1}H NMR, COSY (300 MHz, benzene-*d6*, δ): 3.95–3.94 (d, *J* = 2.4 Hz, 2H, -OCH_2CCH), 3.77–3.24 (m, 374H, polyether backbone and glycerol H), 2.13–2.12 (t, *J* = 2.4 Hz, 1H, -OCH_2CCH), 1.69–1.57 (m, 12H, -OCH_2CH_2-), 1.47–1.30 (m, 60H, -$OCH_2CH_2(CH_2)_{15}CH_3$), 0.94–0.90 (m, 6H, -$O(CH_2)_{17}CH_3$).

S2.5. Synthesis of 1,2-Bis-N-Octadecyl Glyceryl Ether (BisHD)

The synthesis was carried out according to literature.[2]

^{1}H NMR, COSY (400 MHz, chloroform-*d*, δ): 3.78–3.36 (m, 9H, glycerol H), 1.63–1.49 (m, 4H, -OCH_2CH_2-), 1.40–1.17 (m, 52H, -$OCH_2CH_2(CH_2)_{13}CH_3$), 0.88 (t, *J* = 13.1 Hz; 7.2 Hz, 6H; -$O(CH_2)_{17}CH_3$).

S2.6. Synthesis of 1,2-Bis-N-Icosanyl Glyceryl Ether (BisID)

The synthesis was carried out according to literature.[3]

Dry tetrahydrofuran (THF) was placed in three-necked round bottom flask equipped with Dimroth condenser and sealed precision glass (KPG) stirrer. Under argon atmosphere and stirring 3-benzyloxy-1,2-propanediol (2.7 mL, 0.017 mol, 1 eq.), sodium hydride (1.62 g, 0.068 mol, 4 eq.) and 1-bromoicosane (24.42 g, 0.068 mol, 4 eq.) was added. The reaction mixture was stirred at 80 °C for 9 days. Additional NaH was added (1.00 g, 0.042 mol, 2.5 eq) and the solution was stirred for another 23 days. The solvent was removed under reduced pressure to obtain a total volume of 250 mL. Water

(250 mL) and diethyl ether (250 mL) was added and the mixture was stirred overnight at room temperature. To neutralize the reaction mixture, sulfuric acid (1 mol·L^{-1}, 15 mL, 0.015 mol) was added and again stirred overnight. The organic phase was extracted three times with diethyl ether (150 mL each) and dried over sodium sulfate. The solvent was removed under reduced pressure and the crude product was purified using flash column chromatography (petroleum ether/diethyl ether 40:1). The intermediate 1,2-bis-*n*-icosanyl-3-benzyl glyceryl ether (4.97 g, 0.007 mol) was obtained as colorless solid. Yield: 41%.

1,2-Bis-*n*-icosanyl-3-benzyl glyceryl ether (4.97 g, 0.007 mol, 1 eq.) was dissolved in dichloromethane. Palladium on activated charcoal was added (5 weight percent). Hydrogen was introduced and the mixture was stirred at room temperature for 20 days. The catalyst was removed via filtration over celite®. Afterwards the solvent was removed under reduced pressure to obtain 1,2-bis-*n*-icosanyl glyceryl ether (1.84 g, 0.003 mol) as colorless solid. Yield: 42%.

^{1}H NMR, COSY (400 MHz, chloroform-*d*, δ): 3.74–3.41 (m, 9H, glycerol H), 1.60–1.52 (m, 4H, -OCH_2CH_2-), 1.34–1.21 (m, 68H, -$OCH_2CH_2(CH_2)_{17}CH_3$), 0.89–0.86 (m, 6H, -$O(CH_2)_{19}CH_3$).

^{13}C NMR, HSQC, HMBC (101 MHz, chloroform-*d*, δ): 78.4 (glycerol C), 72.0 (-OCH_2CH_2-), 71.1 (glycerol C), 70.6 (-OCH_2CH_2-), 63.25 (glycerol C), 32.1 (-$OCH_2CH_2(CH_2)_{17}CH_3$), 30.2 (-OCH_2CH_2-), 29.9–29.5 (-$OCH_2CH_2(CH_2)_{17}CH_3$), 26.3 (-$OCH_2CH_2(CH_2)_{17}CH_3$), 22.9 (-$OCH_2CH_2(CH_2)_{17}CH_3$), 14.3 (-$O(CH_2)_{19}CH_3$).

S2.7. Polymer Synthesis of BisID-PEG

The synthesis is described for BisID-PEG_{62} as a representative example.

1,2-Bis-*n*-icosanyl glyceryl ether (BisID) (0.2 g, 0.307 mmol, 1 eq.) was placed in a dry Schlenk flask and dissolved in benzene (10 mL). The solution was stirred at 60 °C for at least 30 min and dried in vacuo for 16 h to remove moisture. Dry tetrahydrofuran (approx. 10 mL) was cryo-transferred to the Schlenk flask to dissolve the initiator. Afterwards, the solution was stirred and the initiator was deprotonated with a 0.5 M solution of potassium naphthalenide in THF (0.31 mL, 0.153 mmol, 0.5 eq.). The solution was cooled down to –90 °C and ethylene oxide (EO) (0.95 mL, 20.919 mmol, 68 eq.) was cryo-transferred using a graduated ampule. The polymerization was carried out at 60 °C for 24 h. Subsequently, the reaction mixture was heated to 80 °C for 16 h. To quench the polymerization, an excess of ethanol was added and the solvent was removed under reduced pressure. The crude product was dissolved in methanol and precipitated three times in cold diethyl ether to obtain the pure product. Yield: 95%.

^{1}H NMR, COSY (400 MHz, benzene-*d_6*, δ): 3.73–3.31 (m, 256H, polyether backbone and glycerol H), 1.67–1.58 (m, 4H, -OCH_2CH_2-), 1.45–1.27 (m, 70H, -$OCH_2CH_2(CH_2)_{15}CH_3$), 0.93–0.89 (m, 6H, -$O(CH_2)_{19}CH_3$).

^{13}C NMR, HSQC, HMBC (101 MHz, benzene-*d_6*, δ): 79.0 (glycerol C), 73.4 (polyether backbone and glycerol C), 72.4–70.8 (polyether backbone and glycerol C), 62.1 (polyether backbone and glycerol C), 32.6 (-$OCH_2CH_2(CH_2)_{16}CH_2CH_3$), 31.1–30.1 (-$OCH_2CH_2$- and -$OCH_2CH_2(CH_2)_{16}CH_2CH_3$ (BisID)), 27.0 (-$OCH_2CH_2(CH_2)_{16}CH_2CH_3$ (BisID)), 23.4 (-$OCH_2CH_2(CH_2)_{16}CH_2CH_3$ (BisID)), 14.6 (-$O(CH_2)_{19}CH_3$ (BisID)).

S2.8. Functionalization of BisID-PEG with Propargyl Bromide

The functionalization is described for BisID-PEG_{62}-alkyne as a representative example.

BisID-PEG_{62} (0.2 g, 0.059 mmol, 1 eq.) was placed in a Schlenk flask and dissolved in dry THF (10 mL). The solution was cooled to 0 °C and sodium hydride (0.004 g, 0.178 mmol, 3 eq.) was added. Afterwards, propargyl bromide (0.016 mL, 0.178 mmol, 3 eq.) was added, and the solution was stirred for 24 h at room temperature. The reaction mixture was filtered, and the solvent was reduced under reduced pressure. The remaining solution was precipitated twice in cold diethyl ether and the pure product was dried in vacuo. Yield: 61%.

^{1}H NMR, COSY (300 MHz, benzene-*d6*, δ): 3.95–3.94 (d, *J* = 2.4 Hz, 2H, -OCH_2CCH), 3.77–3.24 (m, 245H, polyether backbone and glycerol H), 2.12–2.11 (t, *J* = 2.4 Hz, 1H, -OCH$_2$CC*H*), 1.69–1.57 (m, 4H, -OCH$_2$CH_2-), 1.49–1.28 (m, 67H, -OCH$_2$CH$_2$(CH_2)$_{15}$CH$_3$), 0.94–0.90 (m, 6H, -O(CH$_2$)$_{17}$CH_3).

Scheme S1: Synthesis Route of the Dialkyl PEG-Lipids and Functionalization with propargyl bromide.

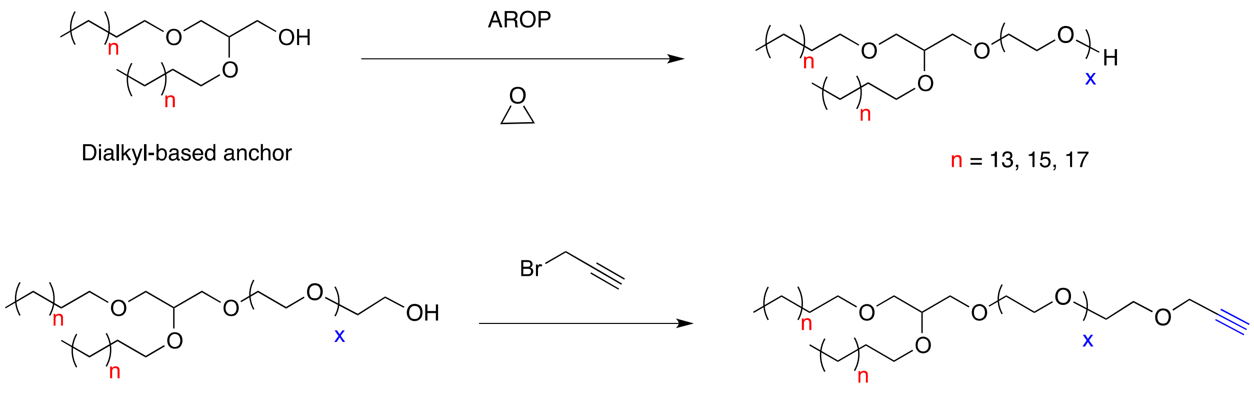

S2.9. Polymer Synthesis of Cholesterol-PEG-PEEGE

The synthesis protocol was described in previous reports.[3]

^{1}H NMR, COSY (300 MHz, DMSO-*d6*, δ): = 5.30 (C=CH cholesterol), 4.63 (br, CHO acetal), 3.72–3.21 (polyether backbone; CHO cholesterol), 2.28–0.82 (br, CH_2, CH cholesterol), 1.18–1.06 (br, CH_3 acetal), 0.64 (br, CH_3 cholesterol).

S2.10. Polymer Synthesis of Cholesterol-PEG-linPG

The synthesis protocol was described in previous reports.[3]

^{1}H NMR, COSY (300 MHz, DMSO-*d6*, δ): = 5.30 (C=CH cholesterol), 4.24 (br, OH), 3.72–3.21 (polyether backbone; CHO cholesterol), 2.28–0.82 (br, CH_2, CH cholesterol), 0.64 (br, CH_3 cholesterol).

S2.11. Polymer Synthesis of Cholesterol-PEG-hbPG

The synthesis protocol was described in previous reports.[3]

^{1}H NMR, COSY (300 MHz, DMSO-*d6*, δ): = 5.30 (C=CH cholesterol), 4.77–4.43 (br, OH, different signals due to hyperbranched PG), 3.76–3.22 (polyether backbone; CHO cholesterol), 2.28–0.82 (br, CH_2, CH cholesterol), 0.64 (br, CH_3 cholesterol).

S2.12. Functionalization of Cholesterol-PEG-hbPG with Propargyl Bromide

The synthesis protocol was described in previous reports.[3]

^{1}H NMR, COSY (300 MHz, DMSO-*d6*, δ): = 5.30 (C=CH cholesterol), 4.77–4.43 (br, OH, different signals due to hyperbranched PG), 4.31–4.17 (OCH$_2$C≡CH), 3.76–3.22 (polyether backbone; CHO cholesterol), 2.45 (C≡CH), 2.28–0.82 (br, CH_2, CH cholesterol), 0.63 (br, CH_3 cholesterol).

Scheme S2: Synthesis Route of the Cholesterol-PEG-hbPEG Lipids and Functionalization with propargyl bromide.

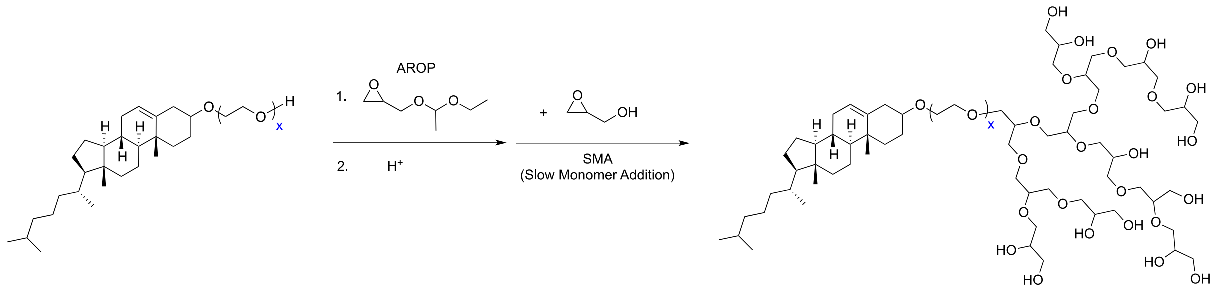

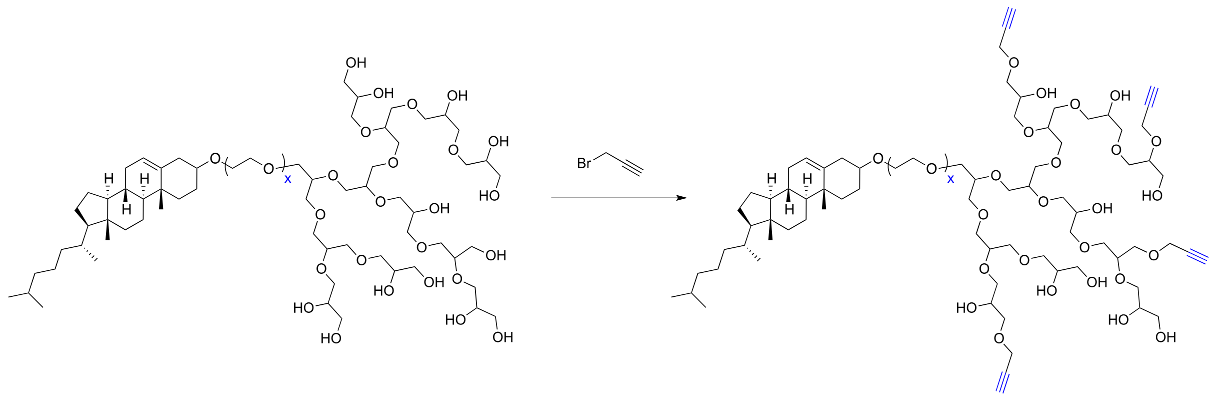

References

1. Fitton, A. O.; Hill, J.; Jane, D. E.; Millar, R. Synthesis of Simple Oxetanes Carrying Reactive 2-Substituents. *Synthesis* **1987**, 1140–1142, doi:10.1055/s-1987-28203.
2. Stauch, O.; Uhlmann, T.; Fröhlich, M.; Thomann, R.; El-Badry, M.; Kim, Y.-K.; Schubert, R. Mimicking a Cytoskeleton by Coupling Poly(*N*-isopropylacrylamide) to the Inner Leaflet of Liposomal Membranes: Effects of Photopolymerization on Vesicle Shape and Polymer Architecture. *Biomacromolecules* **2002**, *3*, 324–332, doi:10.1021/bm015613y.
3. Hofmann, A. M.; Wurm, F.; Frey, H. Rapid Acess to Polyfunctional Lipids with Complex Architecture via Oxyanionic Ring-Opening Polymerization. *Macromolecules* **2011**, *44*, 4648–4657, doi:10.1021/ma200367c.
